# Intravitreal Ranibizumab Injections Significantly Influence Retinal Venous Calibre in Patients With Diabetic Macular Oedema

**DOI:** 10.1155/joph/5637071

**Published:** 2025-12-18

**Authors:** Luke K. Chehade, Noha Ali, Paul G. Sanfilippo, Sukhpal S. Sandhu, Lyndell L. Lim, Sanjeewa S. Wickremasinghe

**Affiliations:** ^1^ Ophthalmology Department, University of Adelaide, Adelaide, South Australia, Australia, adelaide.edu.au; ^2^ Centre for Eye Research Australia Medical Retina Department, The Royal Victorian Eye and Ear Hospital, Melbourne, Victoria, Australia; ^3^ Cerulea Clinical Trials Medical Retina Department, The Royal Victorian Eye and Ear Hospital, Melbourne, Victoria, Australia; ^4^ Ophthalmology Department, Assuit University, Assuit, Egypt, aun.edu.eg; ^5^ University of Melbourne Ophthalmology Department, Melbourne, Victoria, Australia

**Keywords:** macular oedema, retinal vessels, vascular endothelial growth factor

## Abstract

**Purpose:**

To assess changes in retinal vascular calibre following intravitreal ranibizumab treatment in patients with diabetic macular oedema (DMO).

**Methods:**

A post hoc analysis of data from a prospective clinical study assessing patients treated with ranibizumab for DMO was conducted. 76 eyes of 67 treatment naive patients were recruited. Patients received three, monthly, ranibizumab injections. From months 3 to 12, ranibizumab injections were administered PRN. Retinal vascular calibre was measured from digital fundus photographs with a semiautomated computer programme (SIVA) and summarised as central retinal artery (CRAE) and vein (CRVE) equivalent. Artery to vein ratio (AVR) was derived from CRAE/CRVE. The primary and secondary outcomes were change in CRAE, CRVE, AVR, CMT and BCVA from baseline to month 2 and 12, respectively.

**Results:**

BCVA improved significantly from 60.71 ± 8.24 letters by 6.76 ± 6.40 (*P* < 0.001) at month 2 and 10.12 ± 10.86 (*P* < .001) at 12. CMT, 470.58 μm ± 106.91 μm, significantly reduced by 81.41 μm ± 99.73 μm (*P*  < 0.001) at month 2 and 132.48 ± 141.79 μm (*P* < 0.001) at month 12. There was a statistically significant CRVE decrease by −8.44 μm ± 11.27 μm (*P* < 0.0001) at month 2 and −4.68 ± 12.41 μm (*P* = 0.03) at month 12, whilst AVR increased by +0.02 μm ± 0.04 μm (*P* = 0.02) at month 2 and +0.02 μm ± 0.04 μm (*P* = 0.01) at month 12.

**Conclusion:**

Ranibizumab treatment was associated with a significant reduction in CRVE and increase in AVR. It is possible that the positive effect of ranibizumab therapy on improvements in BCVA and CMT may be related to reduction of retinal venous calibre and hydrostatic pressure.

## 1. Introduction

Diabetic retinopathy (DR) is the most common cause of legal blindness in working age individuals. Diabetic macular oedema (DMO) is a serious complication of DR that leads to severe visual loss [1, 2]. DMO is thought to be a consequence of increased retinal capillary permeability which results from endothelial cell dysfunction, thickening of the retinal capillary basement membrane and decreased numbers of the pericytes. Although the mechanisms of these retinal changes are not fully understood, there are both mechanical components related to vascular endothelial dysfunction and hydrostatic pressure, as well as inflammatory and angiogenic mediated mechanisms which play a role in the pathogenesis of both DR and DMO [3–8].

DMO is a consequence of both cytotoxic and vasogenic mechanisms. Cytotoxic oedema largely occurs due to hyperglycaemia and the resultant intracellular buildup of sorbitol, lactate and phosphates, and this is the predominant mechanism early in the disease course. Vasogenic oedema results from rupture of the inner blood–retinal barrier (i‐BRB) secondary to elevated levels of VEGF, nitrous oxide and free radicals. The imbalance between fluid buildup and its resorption via dysfunctional Müller cell metabolism results in DMO [9–11].

The predominance of intravitreal anti‐VEGF as the initial treatment choice for DMO underscores the importance of VEGF in the pathophysiology of DMO [12], but conversely its ineffectiveness in a portion of patients highlights the multifactorial nature of DMO and the need to understand and target other inflammatory and angiogenic cytokines. Inflammatory as well as angiogenic cytokines are known to be elevated within the blood and aqueous of eyes with proliferative and severe nonproliferative diabetic retinopathy (NPDR) compared to control eyes without diabetes [6–8]. Chronic hyperglycemia induced expression of VEGF‐A, placenta growth factor (PlGF), interleukin‐8 (IL‐8), IL‐6, IL‐1β, TNF‐α and MMPs, which have all been implicated in disruption of the BRB [13], a key component in DMO pathophysiology.

VEGF has been shown to induce retinal vasodilation through the increased expression of endothelial nitric oxide, which is a potent vasodilator [14], and previous epidemiological studies have found larger arteriolar and venular calibre in patients with diabetes and in those with impaired glucose tolerance [15–19]. In addition, larger venular calibre linked with increasing severity of DR is also considered as a systemic marker of inflammation [20]. It has been speculated that larger vessels may lead to increased capillary blood flow and increased fluid leakage from the retinal capillaries with increased hydrostatic pressure [21].

Several studies have evaluated changes in vascular calibre post intravitreal steroids and anti‐VEGF in eyes with macular oedema secondary to retinal vein occlusions (RVOs) or DMO [22–30]. A number of these studies have demonstrated reduction in venular calibre following intravitreal steroid injection for DMO [24–26, 28] and RVO [22]. Evidence for changes in vessel calibre following intravitreal anti‐VEGF is less convincing, with one study demonstrating reduction in vessel calibre in eyes with RVO [24], and one study demonstrating reduction in both central retinal artery equivalent (CRAE) and central retinal vein equivalent (CRVE) after a single injection [30], but many others failing to confirm this [23, 26, 27, 29].

Finally, in a small study of 25 patients, wider baseline retinal venular calibre was associated with better visual improvement following intravitreal ranibizumab for DMO, further highlighting the importance of vessel calibre in this condition [31].

Given the disparity in findings amongst previous studies, as well the potential role of vessel calibre as a predictor for visual improvement, we proposed to examine this further in a post hoc analysis of data from a prospective study [32] evaluating the effect of intravitreal ranibizumab injections on aqueous concentrations of angiogenic or inflammatory cytokines in patients with centre‐involved DMO. As part of the study, retinal vascular calibre was assessed at baseline, 2 months (after 2 of 3 loading injections) and 12 months. All patients received 3, monthly loading injections of ranibizumab, followed by ‘as required’ injections. We therefore examined the changes in retinal vascular calibre between these three time points.

## 2. Methods

### 2.1. Population

Subjects were enroled from the retina clinics of Royal Victorian Eye and Ear Hospital in Melbourne as part of a study to evaluate the effect of intravitreal ranibizumab injections on aqueous concentrations of angiogenic or inflammatory cytokines in patients with DMO. Recruitment occurred over two time periods. An initial group of 25 patients (30 eyes) were enroled from June 2015 until May 2016 as part of the DISCERN study [32–34]. Subsequently, a further 42 patients (46 eyes) were enroled from May 2018 until July 2021. The study was conducted in accordance with the tenets of the Declaration of Helsinki.

Retinal calibre measurements were analysed in this group of patients in this post hoc analysis.

The recruitment criteria and treatment protocols have been detailed previously [32–34], but in brief, all patients having centre‐involving DMO had a best‐corrected visual acuity (BCVA) of 17–72 logMAR (logarithm of the minimum angle of resolution) letters (Snellen equivalent, 20/400 to 20/40). Each patient received ranibizumab treatment as per the RESTORE study protocol [35], with three, monthly loading dose injections followed by *PRN* treatment to 12 months.

### 2.2. Measurement of Retinal Vascular Calibre

At baseline, 2 and 12 months, fundus photographs were performed using a standardised protocol. Images centred on the optic disc (ETDRS Field 1) were used to measure retinal vascular calibre with a semiautomated computer programme (Singapore ‘I’ Vessel Analysis‐SIVA, National University of Singapore) based on a modified protocol. SIVA automatically identifies the optic disc, places a grid with reference to the centre of optic disc, identifies vessel type and measures retinal vascular calibre.

SIVA measures the largest six arterioles and largest six venules coursing through an area one‐half to one‐disc diameter from the optic disc margin. These individual measurements are summarised as the CRAE and CRVE, respectively, using the revised Knudtson–Parr–Hubbard formula [36]. Studies investigated intragrader reliability on 200 randomly selected retinal photographs, and intraclass correlation coefficient (95% confidence interval) was 0.99 (0.98–0.99) for CRAE and 0.94 (0.92–0.96) for CRVE, respectively [37].

### 2.3. Statistical Methods

Data management and statistical tests were performed in the R statistical environment (version 4.3.0, R Development Core Team 2024). Continuous and categorical outcomes were reported as means ± standard deviation and frequencies (percentages), respectively. Subjects with centre‐involving DMO and gradable retinal photographs from at least one measured time point (baseline, month 2 and month 12) were considered for analysis. The primary outcomes of interest were the change in vascular parameters (CRAE, CRVE and AVR), CMT and BCVA from baseline to month 2. The primary analysis set therefore consisted only of subjects with complete data at both baseline and month 2. Secondary outcomes were changes in the same parameters from baseline to month 12. Statistical tests of change in each parameter from baseline to month 2 (and baseline to month 12) were performed with a one‐sample *t*‐test against the null hypothesis of no change. Baseline comparisons of parameters (age, gender, etc.) across subjects with complete versus missing data on the primary outcomes were conducted with either independent samples *t*‐tests or chi‐square tests as appropriate. All statistical tests were two‐sided, with a *p* value of less than 0.05 considered statistically significant.

## 3. Results

At baseline, 76 eyes of 67 patients (49 men and 18 women) with centre‐involving DMO were recruited. Of these 76 eyes, 59 (77.6%) had gradable retinal photographs at baseline; 40 (52.6%) at month 2 and 39 (51.3%) at month 12. In total, 35 (46.1%) eyes had retinal vessel images available at baseline, month 2 and month 12 and were included in the analysis as ‘Study’ eyes. The fellow eyes of recruited patients that were untreated were analysed as ‘nonstudy’ eyes. Of these eyes, 40 (68.9%) had gradable photographs at baseline and 29 (50.0%) had gradable images at month 2, whilst 25 (58.0%) had gradable images at month 12. Retinal vascular assessment was not possible in excluded eyes because of inadequate disc centration of the photographs, media opacity or poor‐quality images. There were no significant differences in gender, baseline CMT, baseline blood pressure, DRSS, HbA1C or duration of diabetes between those included compared to those excluded within the study eyes (based on photographic images). Those without images however were older (66.7 ± 8.9 vs. 62.2 ± 8.3 years, *P* = 0.02), and had slightly worse BCVA (57.71 ± 9.83 compared with 61.97 ± 7.29, *P* = 0.03). The baseline characteristics of the treated eyes are shown in Table 1. There was also no statistically significant difference in the final BCVA (*P* > 0.05) or CMT (*P*  >  0.05) between the included and excluded eyes.

**Table 1:** Baseline characteristics of study eyes with centre‐involved diabetic macular oedema.

Baseline characteristics	Study eyes (*n* = 76)		*p*
	Included^†^ (*n* = 35)	Excluded (*n* = 41)	
Age	62.20 ± 8.32	66.73 ± 8.89	0.02^∗^
Sex, male	24 (68.6%)	30 (73.2%)	0.85
History of hypertension	26 (74.3%)	27 (65.9%)	0.58
Duration of diabetes	14.48 ± 8.82	15.44 ± 7.40	0.61
HbA1c	8.18 ± 1.61	8.43 ± 2.13	0.56
DRSS (retinopathy score)	53.03 ± 11.89	50.18 ± 13.10	0.34
Baseline BCVA (letters)	61.97 ± 7.29	57.71 ± 9.83	0.03^∗^
Baseline CMT	464.54 ± 104.54	495.66 ± 122.02	0.24
Baseline systolic BP	145.57 ± 17.06	145.10 ± 18.67	0.91
Baseline diastolic BP	86.51 ± 9.36	83.17 ± 10.21	0.14

*Note:* Data are expressed as mean (SD) or count (%). *P* relates to test of proportions from chi‐square test or test of equality of means from independent *t*‐test as appropriate.

^∗^Statistically significant.

^†^Complete baseline and month 2 data.

All eyes received ranibizumab treatment at baseline, month 1 and month 2, and over the total period from baseline to month 12, 7.9 ± 3.2 injections were administered (range 3–13).

Table 2 shows the changes in BCVA, CMT and retinal vascular parameters from baseline to month 2 and month 12. BCVA improved significantly from baseline 60.71 ± 8.24 letters by 6.76 ± 6.40 (*P* < 0.001) letters by month 2 and 10.12 ± 10.86 (*P* < .001) letters by month 12. Moreover, the CMT significantly reduced from baseline 470.58 μm ± 106.91 μm by 81.41 μm ± 99.73 μm (*P*  <  0.001) at month 2 and −132.48 ± 141.79 μm (*P* < 0.001) at month 12.

**Table 2:** Visual acuity, central macular thickness and retinal vascular calibre at baseline and after ranibizumab injections^∗∗^.

Parameter	Baseline	Change at month 2	*P*	Change at month 12	*P*
CRAE	142.25 ± 14.43	−1.87 ± 6.63	0.11	1.10 ± 9.43	0.50
CRVE	215.25 ± 24.87	−8.44 ± 11.27	< 0.001^∗^	−4.68 ± 12.41	0.03^∗^
AVR	0.67 ± 0.07	0.02 ± 0.04	0.02^∗^	0.02 ± 0.04	0.01^∗^
CMT	470.58 ± 106.91	−81.41 ± 99.73	< 0.001^∗^	−132.48 ± 141.79	< 0.001^∗^
BCVA	60.71 ± 8.24	6.76 ± 6.40	< 0.001^∗^	10.12 ± 10.86	< 0.001^∗^

*Note:* Data are expressed as mean and SD. Statistically analysed with one sample​ *t*‐test. Change score is calculated as month 2/12 score minus baseline score.

Abbreviations: AVR, artery to vein ratio; BCVA, best‐corrected visual acuity; CMT, central macular thickness; CRAE, central retinal artery equivalent; CRVE, central retinal vein equivalent.

^∗^Statistically significant.

^∗∗^At 2 months, all eyes had received 2, monthly ranibizumab injections, whilst at 12 months, all eyes had received 3 monthly ranibizumab injections followed by ‘as required’ injections as per the RESTORE study protocol.

There was a marginal, nonsignificant decrease in CRAE by −1.87 μm ± 6.63 μm (*P* = 0.11) at month 2 but no change was noted by month 12, +1.10 μm ± 9.43 μm (*P* = 0.50). There was a statistically significant CRVE decrease by −8.44 μm ± 11.27 μm (*P* < 0.0001) at month 2 and −4.68 ± 12.41 μm (*P* = 0.03) at month 12, whilst the AVR increased slightly by +0.02 μm ± 0.04 μm (*P* = 0.02) at month 2 and +0.02 μm ± 0.04 μm (*P* = 0.01) at month 12. The baseline, month 2 and month 12 retinal vascular parameters are depicted in Figure 1.

Figure 1(a–c) Change in vascular parameters from baseline to month 2 and month 12 in study (included) versus nonstudy (untreated fellow) eyes with 95% CI.

**Figure:**
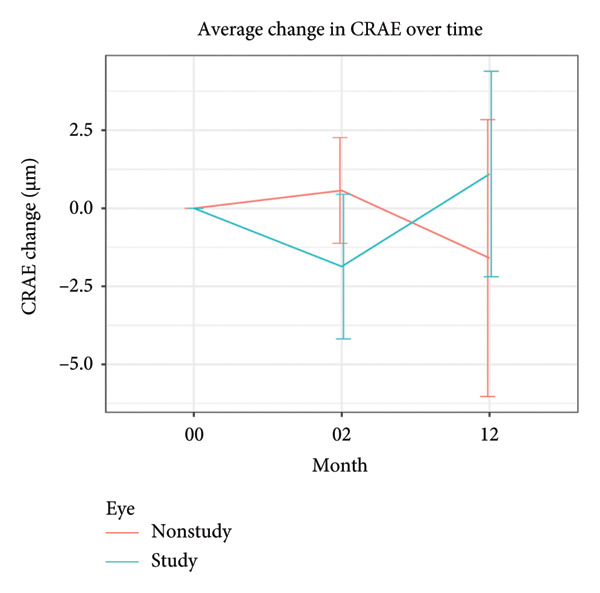
(a)

**Figure:**
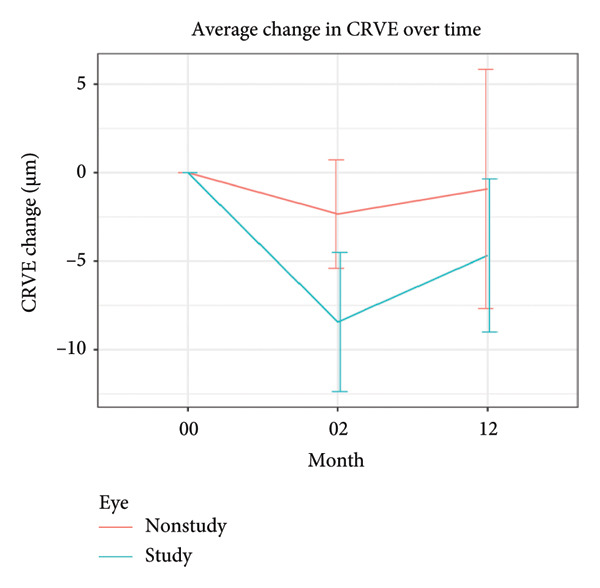
(b)

**Figure:**
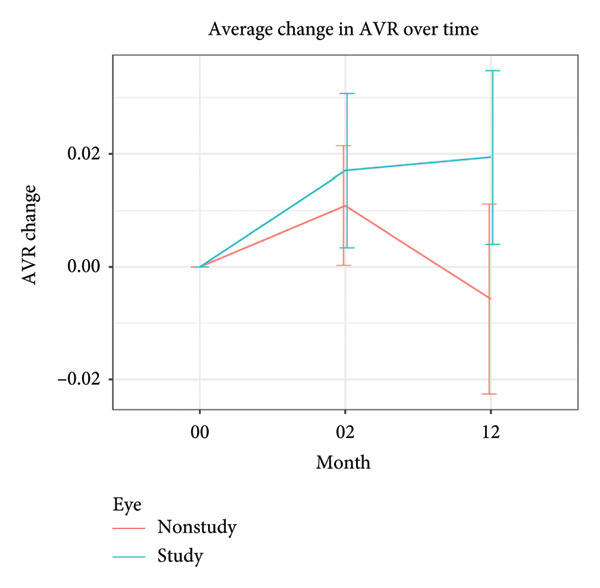
(c)

Baseline CRAE, CRVE or AVR did not correlate with BCVA or CMT at baseline, month 2 or month 12 (*P* > 0.05). Additionally, baseline CRAE, CRVE or AVR were not associated with improvements in BCVA or CMT at either month 2 or month 12 (*P* > 0.05). The number of injections administered by month 12 was not associated with BCVA or CMT at month 12 (*P* > 0.05).

There was however a significant reduction in both systolic blood pressure (145.57 mmHg ± 17.06 mmHg) at the baseline decreasing by 11.39 mmHg ± 18.44 mmHg after 2 months (*P* = 0.001) and diastolic blood pressure (86.51 mmHg ± 9.34 mmHg) at the baseline decreasing by 3.73 mmHg ± 10.71 mmHg (*P* = 0.05) at month 2. There was no correlation between reduction in systolic (*P* = 0.94) or diastolic (*P* = 0.90) blood pressure and reduction in CRVE.

## 4. Discussion

In this post hoc analysis, we investigated the effect of intravitreal ranibizumab injections on retinal vessel calibre in patients with DMO [32]. We found that ranibizumab was associated with a significant reduction in retinal venous calibre at both month 2 and month 12, as well as increase in central retinal artery to vein ratio.

Previous population‐based studies have demonstrated changes in CRVE and CRAE in patients with DR, as well as the effect of associated systemic risk factors on these changes [17]. Analysis of retinal images from 3654 patients in the Blue Mountain Eye Study demonstrated significantly increased retinal venular calibre in diabetic patients compared with nondiabetic patients (262.7 μm in severe NPDR compared with 221.9 μm). Mean retinal arteriolar calibre was also significantly wider in patients with diabetes than those without diabetes, although the difference was not as impressive (201.1 μm in severe NPDR compared with 190.2 μm in nondiabetic patients) [38]. Furthermore, both retinal arterioles and venules in diabetic patients that go on to develop DMO seem to gradually dilate and elongate, compared with patients without significant DR or DMO, suggesting that Starling’s law applies to the development of DMO [39]. Retinal vascular calibre is maintained by several ocular and systematic mechanisms [21]. These mechanisms seem to be disrupted in patients with diabetes, who have larger vessel calibre than the normal population [40]. The enlargement of the vessel diameter in diabetes is postulated to be due to endothelial dysfunction and inflammation. It has been speculated that in the diabetic retina, hyperglycaemia and hypoxia initiate retinal vasodilatation, leading to vascular hyperperfusion and leakage across the vessel wall due to elevation of the transmural hydrostatic pressure [9].

The concept of hydrostatic pressure and its role in DMO is not novel. Macular laser, which was previously the mainstay of therapy causes photothermal destruction of RPE, reduced oxygen consumption in the outer retina, increased oxygen flux from choroid to inner retina, and finally arteriolar constriction and decreased hydrostatic forces [13]. A reduction in the hydrostatic pressure in capillaries and venules, and subsequent venular constriction, leads to reduced DMO [41].

Ranibizumab is a humanised monoclonal antibody that has affinity to all VEGF isotypes, with isoforms of VEGF‐A being the most important promoters of intraocular neovascularization and hyperpermeability [9]. Anti‐VEGF agents including ranibizumab have been shown to reduce macular thickness as well as improve BCVA [35, 42–44], and the reduction in CMT by −132.48 ± 141.79 μm (*P* < 0.001) and increase in BCVA by 10.12 ± 10.86 (*P* < .001) letters by month 12 in the present study is in keeping with this. Furthermore, our study shows that after two, monthly, ranibizumab injections in eyes with DMO, there is significant reduction in retinal venous calibre and increased central retinal artery to vein ratio. These changes were still statistically significant at 12‐month review, following three, monthly, loading doses and further ‘as required’ injections, as per the RESTORE protocol.

A number of papers to date have analysed changes in retinal vasculature in response to intravitreal anti‐VEGF injections [23, 26, 27, 29, 30, 45] which are summarised in Table 3. Of these, only one group has demonstrated a statistically significant change in both CRVE and CRAE.

**Table 3:** Studies assessing retinal vascular calibre changes following anti‐vascular endothelial growth factor treatment.

	Agent	CRAE (μm)		CRVE (μm)	
		Baseline	Change (time period)	Baseline	Change (time period)
Present study	Ranibizumab	142.25 ± 14.43	+1.10 ± 9.43 (12 months)	215.25	−4.28 ± 12.41 (12 months)^∗^
Soliman et al. [23]	Bevacizumab	N/A	−0.2% (4 months)	N/A	−1.9% (4 months)
Wickremasinghe et al. [26]	Bevacizumab	135.5 ± 17.4	+1.7 ± 16.2 (24 months)	201.4 ± 31.2	−4.3 ± 24.2 (24 months)
Tatlipinar et al. [27]	Bevacizumab	80.2 ± 13	+2.8 (1 month)	111.0 ± 9	1.8 (1 month)
Terai et al. [29]	Ranibizumab	186.25 ± 51.4	−15.69 (3 months)	216.21 ± 25	−4.8 (3 months)
Kurt et al. [30]	Bevacizumab	150.21 ± 18.21	4.32 (1 month)^∗^	211.87 ± 29.06	−6.63 (1 month)^∗^
	Ranibizumab	175.42 ± 31.18	−7.95 (1 month)^∗^	235.29 ± 40.79	−16.93 (1 month)^∗^
Sabaner et al.	Aflibercept	190.48 ± 13.50	−20.01 (3 months)	281.73 ± 21.08	+0.24 (3 months)

*Note:* Data are expressed as mean and SD. Change score is calculated as month 2/12 score minus baseline score. Statistically analysed with one sample​ *t*‐test.

Abbreviations: AVR, artery to vein ratio; BCVA, best‐corrected visual acuity; CMT, central macular thickness; CRAE, central retinal artery equivalent; CRVE, central retinal vein equivalent.

^∗^Statistically significant.

Kurt et al. injected the eyes of 62 patients with DMO with bevacizumab (30 patients) or ranibizumab (32 patients), with the untreated fellow eyes used as controls. Measurements were taken preinjection, and at 1 and 4 weeks postinjection. Vascular diameters were, similar to our study, expressed as the CRAE and CRVE, however using a different semiautomated system (Interactive Vessel Analysis, University of Wisconsin), based on digital retinal imaging. There was a statistically significant reduction in CRVE in the ranibizumab group from 235.29 μm preinjection to 218.36 μm at 4 weeks and in the bevacizumab group from 211.87 μm preinjection to 205.24 μm at 4 weeks. Furthermore, there was a statistically significant reduction in CRAE in the ranibizumab group from 175.42 μm preinjection to 167.47 μm at 4 weeks and in the bevacizumab group from 150.21 μm preinjection to 145.89 μm at 4 weeks. Similar to our study, this change in vessel diameter did not correlate with visual acuity [30].

The only other study to assess CRVE and CRAE changes with ranibizumab was by Terai et al. Thirty eyes with DMO received three loading doses of ranibizumab, 4 weeks apart, and images were taken at baseline and months 1, 2 and 3. Whilst statistical significance was not achieved, there was a trend towards reduction in both parameters, with the CRAE reducing from 186.25 μm to 170.56 μm by month 3, and CRVE reducing from 216.21 μm to 211.41 μm by month 3, suggesting that a more adequately powered study may have shown statistical significance [29].

Sabaner et al. is the only study of note to analyse CRVE and CRAE changes with aflibercept 2 mg rather than ranibizumab or bevacizumab. 29 treatment‐naïve eyes with DMO were injected with 3, monthly, aflibercept injections, with CRAE and CRVE measured at baseline and months 1, 2 and 3. Choroidal thickness, CMT and BCVA were also assessed. Their main finding was a reduction in CRAE from 190.48 μm ± 13.50 μm to 170.47 μm ± 23.69 μm at month 3, which is a similar magnitude to Terai et al.’s group. There was no association between this reduction in CRAE and the improvement in BCVA. Interestingly, they did not find any change in CRVE or AVR [45].

With respect to other intravitreal treatments for DMO, our group compared changes in CRVE and CRAE as measured by SIVA following intravitreal dexamethasone implant or bevacizumab in 44 eyes with DMO. Twenty two eyes were injected with each agent, and there was a statistically significant reduction in CRVE and CRAE by −31.8 μm and −6.1 μm, respectively, in the dexamethasone group at 24 months, but no significant change in the bevacizumab group [26]. There are two potential reasons for these contrasting results compared to our present study. First, the follow‐up imaging was taken 2 years after treatment was commenced, and we can see from our study that even at 12 months the initial reduction in CRVE seen at 2 months had started to reverse. Secondly, bevacizumab was used instead of ranibizumab, and it is possible that this may have led to a reduced effect. This is in keeping with Kurt et al.’s study demonstrating that the CRVE reduction was much less impressive using this agent [30].

Tatlipinar et al. imaged 8 patients with DMO receiving a single intravitreal bevacizumab, at baseline, 1 week and 4 weeks post injection, and demonstrated a trend towards vasoconstriction in both the arterioles and venules at 1 week, from 80.2 μm to 77.8 μm and 111.0 μm to 106.5 μm, respectively, but this was not sustained at the 1‐month point. Rather than using semiautomated computer software as in the aforementioned studies, a manually drawn line across the vessel diameter using the measure function of the IMAGEnet programme was utilised [27]. Similarly, Soliman et al. evaluated 10 consecutive eyes injected with intravitreal bevacizumab at three, 6‐weekly intervals, performing fundus photography and OCT at baseline, 1 month, 2.5 months and 4 months. Like the previously mentioned study, retinal vessel calibre was manually measured, this time using images from the fundus fluorescein angiogram. There was an average relative reduction in arteriole trunk vessel diameter at 4 months by 0.2% compared to baseline, and in the venule trunk diameter by 1.9% compared to baseline [23]. There are a number of reasons why the trends in these papers may have failed to reach statistical significance, compared to our paper. Firstly, there was a small sample size. Secondly, bevacizumab was used. Finally, the measurement technique was fairly crude, which could introduce inaccuracies into the already limited data.

As far as a biomarker for BCVA and CMT improvement, whilst Moradi et al. did not specifically measure change in CRVE or CRAE following intravitreal ranibizumab treatment, they found that baseline vessel calibre was predictive of visual outcome. In 25 eyes of 25 patients with gradable photographs at baseline, eyes with ≥ 2 lines improvement in BCVA following ranibizumab had significantly wider CRVE of 248.3 μm compared to 226.6 μm in the eyes with < 2 lines of BCVA gain. CRAE at baseline was not predictive of visual outcome [31].

Our findings suggest that the significant improvements in both vision and macular thickness that we observed following intravitreal ranibizumab treatments may in part be related to changes in hydrostatic pressure changes within the retinal vasculature, given the reduction in central retinal vein diameter and increase in central retinal artery to vein ratio. The post hoc data analysis did not however show a statistically significant correlation between baseline vascular parameters (CRAE, CRVE or AVR) and BCVA or CMT at baseline or at the end of 12 months of treatment. There was also no statistically significant correlation between number of injections during the PRN period, with BCVA or CMT. As such, baseline vascular calibre does not appear to be a biomarker for visual or anatomic outcomes following anti‐VEGF therapy for DMO, although the number of patients in our study may not have been adequately powered to demonstrate this link.

Our study has some potential limitations. First, the overall number of patients included in this study was small with several eyes with ungradable photographs. Comparing the included and excluded eyes, however, there were no significant differences in baseline characteristics, except for greater age and lower baseline BCVA in the excluded eyes. This could be explained by a greater incidence of senile cataract with increasing age which may have affected the quality of the images. Given the significant proportion of patients with ungradable photographs, future studies should check all measures are taken to optimise image quality including adequate dilation, treatment of any ocular surface disease and allowing adequate time with the patient to ensure quality images are obtained. Second, there was significant decrease in both systolic and diastolic blood pressure by month 2 compared to baseline. Although we cannot fully exclude that this reduction may have influenced the CRVE reduction, multivariate analyses of a large epidemiological study of DR have demonstrated that a −2.06 μm reduction in CRAE and −1.52 μm reduction in CRVE resulted from every 10 mmHg increase in mean arterial blood pressure [17]. Thus, if blood pressure was a significant factor, we may have expected an increase instead of a decrease in CRVE. Furthermore, analysis of our data revealed no correlation between reduction in blood pressure and reduction in CRVE. We speculate that the decrease in the blood pressure by the month 2 visit may be due to a reduction in anxiety levels between time periods, following awareness of the injection procedure and after effects. Finally, though all patients received three loading dose injections by month 2, there were variable numbers of injections administered by month 12 as a result of *PRN* dosing. As a result, patients with more severe disease likely received a greater number of injections.

Whilst not necessarily a limitation, it should be noted that the 2‐month time point was chosen as the images could conveniently be taken just prior to final loading dose that all patients received. Whilst imaging at 3 months may have demonstrated a more significant effect from the full three loading doses, imaging was not available for this time point from the original study, and so this time point could not be used.

In conclusion, our findings show that ranibizumab treatment was associated with a significant reduction in central retinal vein diameter and increase in central retinal artery to vein ratio. This suggests that the beneficial effect of ranibizumab on reducing macular oedema is in part related to reduction of retinal venous calibre and hydrostatic pressure changes. Depending on future studies, CRVE may have a role as an early biomarker to be monitored in retinal vascular disease such as DMO. We hope that the addition of our statistically significant data to the current paucity in this area in the literature will help invigorate further investigation into the role of vessel calibre and its determinants as we continue to search for the optimal treatment for DMO.

## Ethics Statement

This study was approved by the Human Research and Ethics Committee of the Royal Victorian Eye and Ear Hospital (RVEEH), Melbourne, Australia (approval number 13/1123H) as part of the Diabetic macular edema: aqueous and Serum Cytokine profiling to determine the Efficacy of RaNibizumab (DISCERN) study. Research adhered to the tenets of the Declaration of Helsinki. Written informed consent was obtained by a study investigator from all participants prior to enrolment in the study.

## Conflicts of Interest

The authors declare no conflicts of interest.

## Funding

This study was supported by a grant from Novartis Pharma AG. SW (GNT 1128343) and LLL (GNT 1109330) are supported by a National Health and Medical Research Council (NHMRC) Early Career Fellowship. The Centre for Eye Research Australia receives Operational Infrastructure Support from the Victorian Government.

## Data Availability

The data that support the findings of this study are available from the corresponding author upon reasonable request.
